# Early post-surgical recurrence of metastatic vertebral neuro-endocrine tumour treated effectively with chemo-radiotherapy

**DOI:** 10.1051/bmdcn/2019090105

**Published:** 2019-02-22

**Authors:** Arun-Kumar Kaliya-Perumal, Mark Tan, Seh Wee Tee, Shaam Achudan, Wai Ming Yap, Jacob Yoong Leong Oh

**Affiliations:** 1 Department of Orthopaedic Surgery, Spine Division, Tan Tock Seng Hospital Singapore; 2 Department of Orthopaedic Surgery, Melmaruvathur Adhiparasakthi Institute of Medical Sciences and Research Melmaruvathur Tamil Nadu India; 3 Department of Pathology, Tan Tock Seng Hospital Singapore

**Keywords:** Metastasis, Myelopathy, Neuroendocrine tumours, Spinal cord compression, Concurrent chemoradiotherapy

## Abstract

Spinal metastasis of neuro-endocrine tumours (NETs) usually arise from a primary in the lung. We encountered such a patient with NET metastasis to T6 vertebra causing severe cord compression. Considering the neurological status, immediate decompression surgery along with T3-T8 posterior stabilization was done. Early recurrence of the tumour causing near total obliteration of the spinal canal leading to significant neurological compromise was noted within one month of surgery. A second surgery at this stage was avoided due to the risk involved and concurrent chemo-radiotherapy was initiated. The tumour was sensitive to chemo-radiotherapy and rapid resolution was noted on subsequent follow-up visits. With appropriate rehabilitation, patient regained full power to become ambulant with support. This case report highlights a rare, early and aggressive recurrence of metastatic vertebral NET following index surgery which was effectively managed with chemo-radiotherapy.

## 1. Introduction

Neuro-endocrine tumours (NETs) arise from cancerous cells of the widely distributed endocrine system and are considered diverse [1]. The tumour primarily occurs in the gastrointestinal tract or the lung [1–3]. Only 12-22% of patients have metastasis at initial presentation [2]. Spinal metastasis of NETs, which was previously considered rare is becoming increasingly common and is reported to usually arise from a primary lung tumour [4–7]. In this report, we describe a case of newly diagnosed NET, metastasizing from right lung to the T6 vertebra, causing severe cord compression demanding immediate decompression surgery. However, the tumour was highly aggressive in nature and exuberant recurrence was noted within a month after surgery. Such early aggressive recurrence after index surgery effectively responding to chemo-radiotherapy is rarely reported in the literature and is highlighted in this report.

## 2. Case Report

A 45-years-old male engineer with no history of trauma, presented to us with progressive upper back pain for two months that eventually became severe and disabling. The pain was non mechanical and was present even at rest and night. He denied any constitutional symptoms. He had no significant medical history except that he was a smoker for over 20 years.

On neurological examination, patient indicated altered sensation from T8 dermatome and below; however, upper and lower limb motor power was normal. He had an unsteady gait and exaggerated deep tendon reflexes in both lower limbs.

**Fig. 1:**
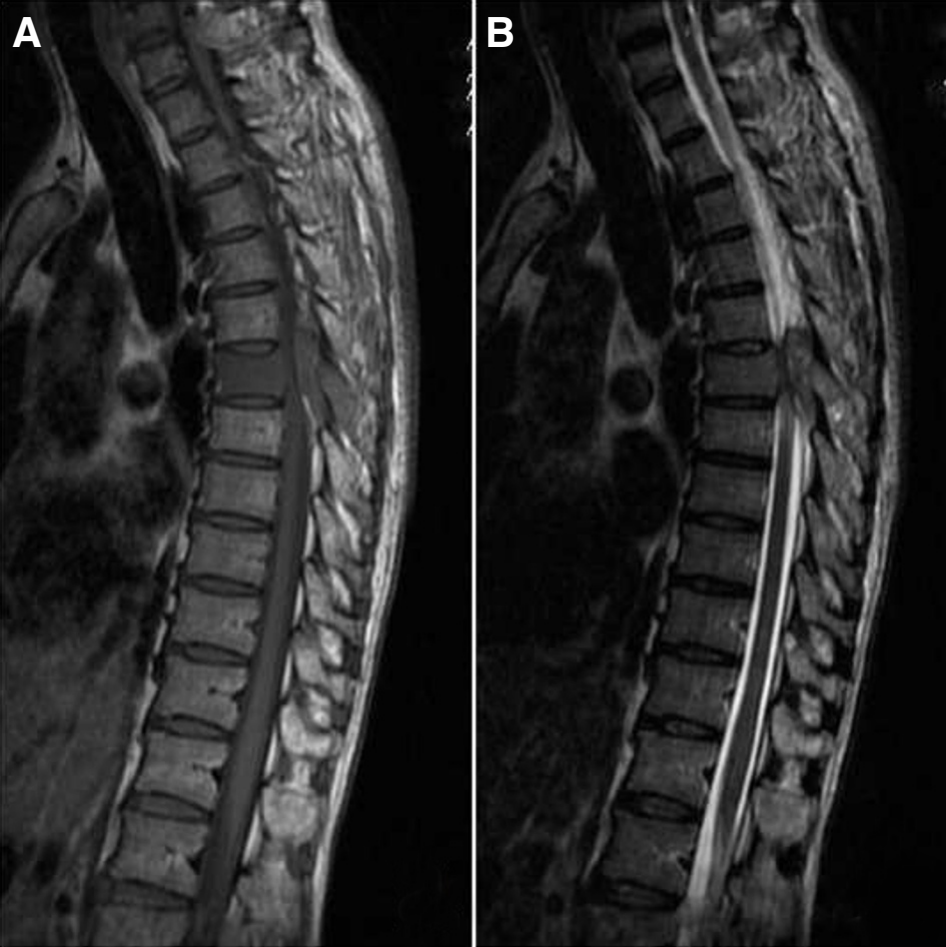
Sagittal MRI images. (A) T1 sagittal cut MRI image showing T6 vertebral involvement. (B) T2 sagittal cut MRI image showing clearly the epidural extension and spinal canal stenosis.

**Fig. 2:**
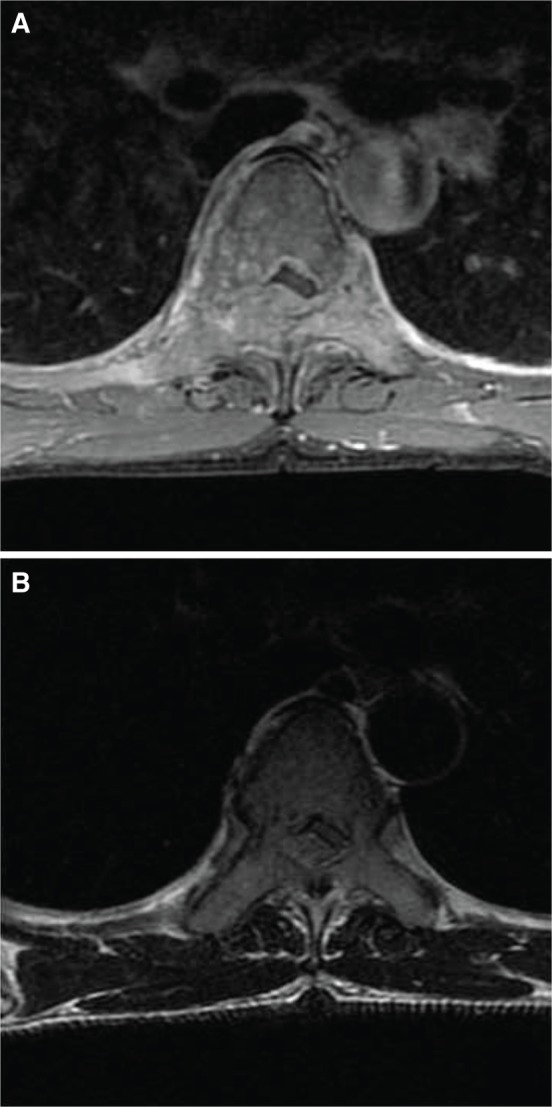
Axial cut MRI images showing the cord compression. (A) Contrast enhanced. (B) T2.

**Fig. 3:**
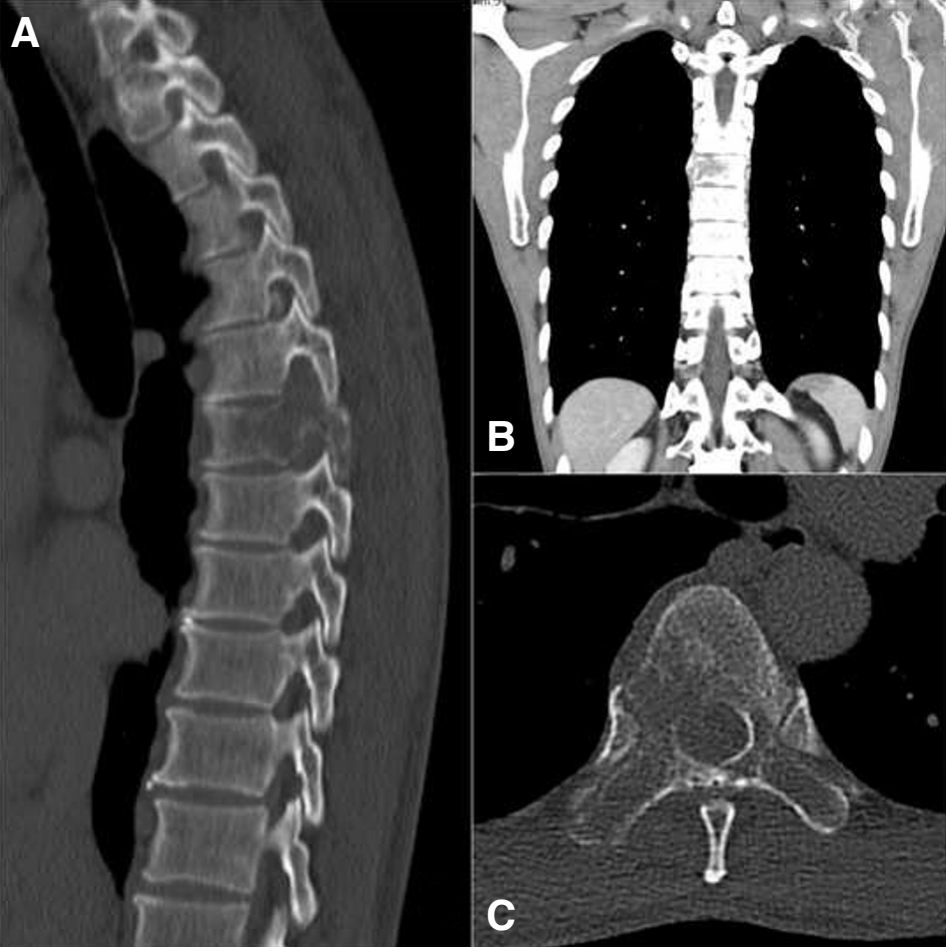
Computerised tomography (CT) images showing the T6 vertebral involvement. (A) Sagittal cut. (B) Coronal cut. view. (B) Lateral view. (C) Axial Cut.

Initial X-rays of the thoracic spine were unremarkable, but MRI demonstrated abnormal marrow replacement and enhancement of the entire T6 vertebra including its posterior elements and right 6th posterior rib. MRI axial cuts at T6 level revealed right postero-lateral epidural extension of the lesion causing severe spinal canal, right lateral recess and right exit foraminal stenosis (Fig. 1, 2). In addition, an irregular right lung nodule was noted. CT evaluation confirmed the vertebral involvement (Fig. 3) and the presence of a lobulated right lung nodule with emphysematous changes in bilateral upper lobes. Further radiological assessment revealed no other lesion elsewhere.

**Fig. 4:**
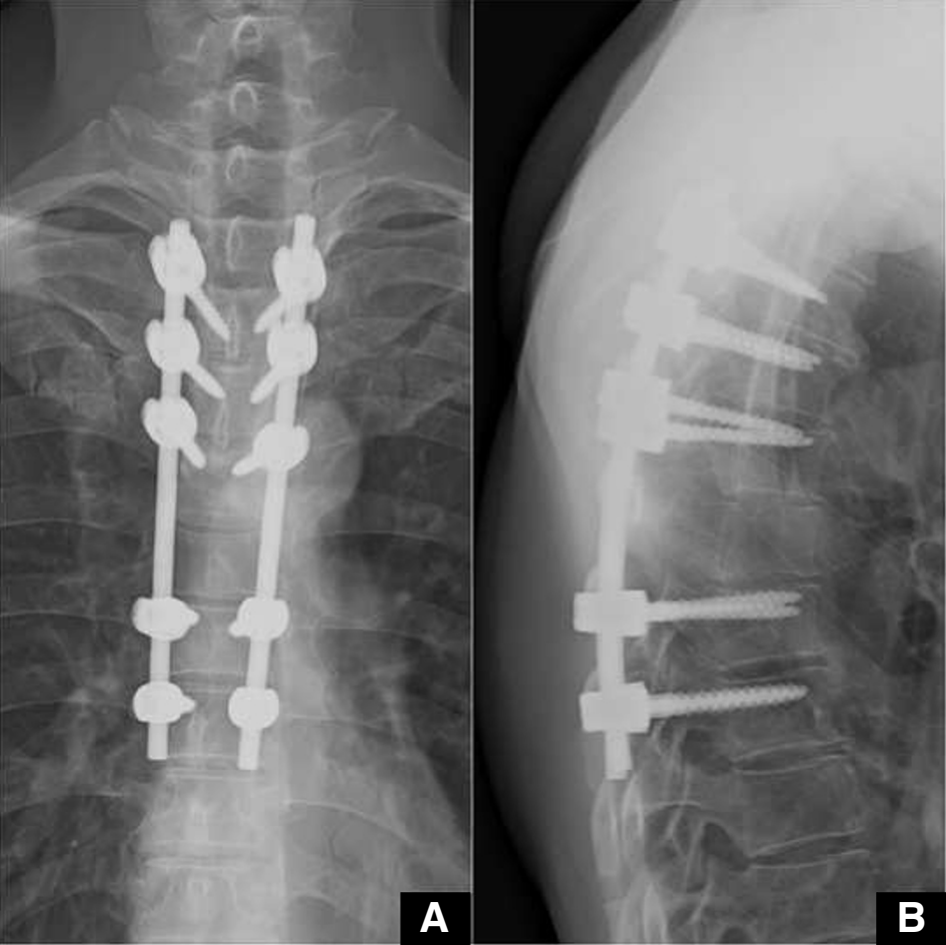
Post-operative X-ray images. (A) Antero-posterior view. (B) Lateral view.

During the course of the work up, he developed bilateral lower limb weakness and was unable to stand. A diagnosis of thoracic myelopathy due to cord compression was made and immediate surgical management in the form of T6 decompression laminectomy along with T3-T8 posterior instrumentation was performed (Fig. 4). Surgery was uneventful; patient had good recovery and was ambulating independently.

**Fig. 5:**
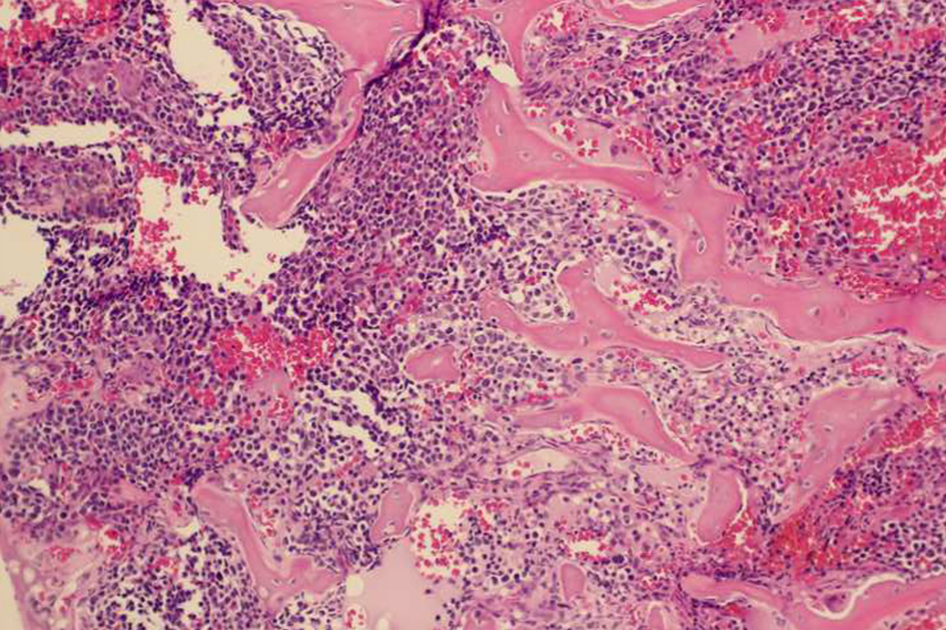
Metastatic tumour cells with scanty to moderate amount of eosinophilic cytoplasm in the marrow spaces. H & E, 100x.

Tissue biopsy from T6 right pedicle revealed presence of metastatic tumour cells with moderate amount of eosinophilic cytoplasm in the marrow spaces suggestive of a metastatic carcinoma with neuro-endocrine features (Fig. 5). Tumour cells were positive for cytokeratin AE1/3, carcinoembrionic antigen (CEA), chromogranin and synaptophysin. Proliferation marker Ki67 was found to be 40%.

Early post-operative MRI showed adequate decompression of the spinal canal at T6 level and the patient had interval resolution of symptoms; but there was abnormal fluid collection from the surgical site extending up to the subcutaneous layer, likely to be seroma, which was conservatively managed. Chemotherapy with Carboplatin and Etoposide was initiated after satisfactory wound healing three weeks after surgery.

**Fig. 6:**
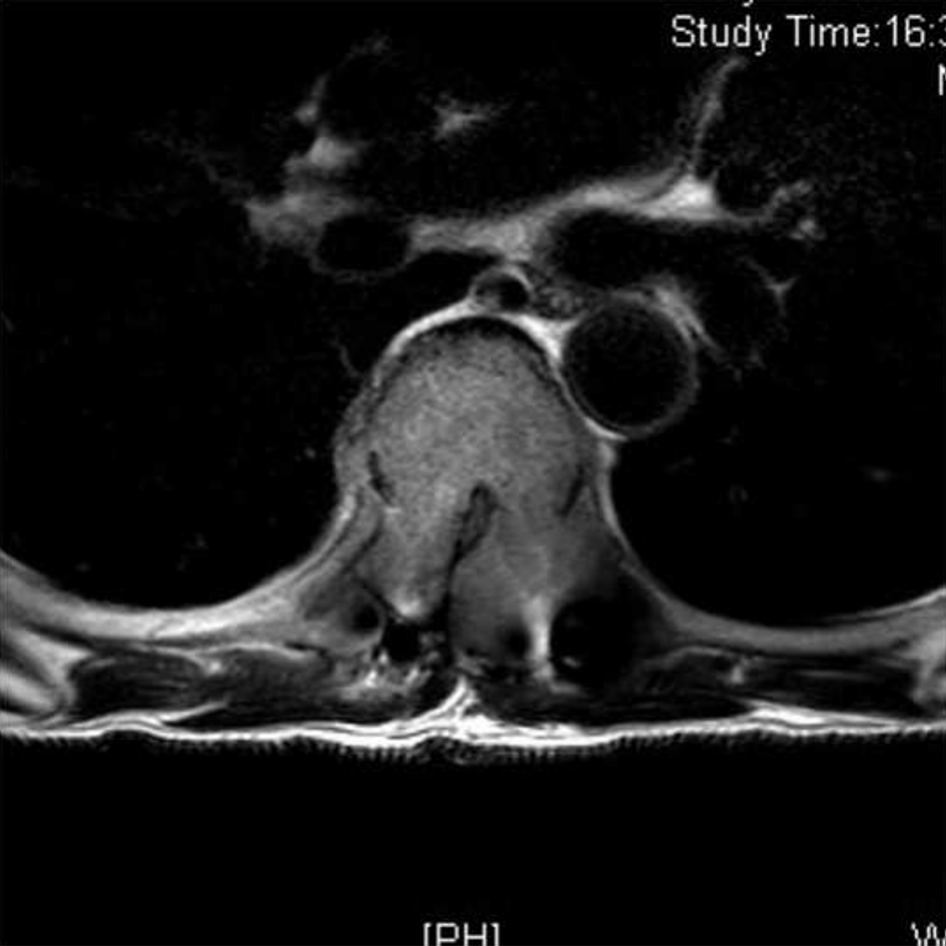
One month post-surgical MRI axial cut image showing near total obliteration of the spinal canal due to the aggressive tumour growth.

One month after the index surgery, patient developed significant motor deficit in the lower limbs (Right L2-L5 = 2/5; Right S1 = 4/5; Left L2-S1 = 4/5) and had bowel and bladder incontinence. An urgent MRI was done which revealed recurrence of the lesion causing near total obliteration of the spinal canal (Fig. 6).

**Fig. 7:**
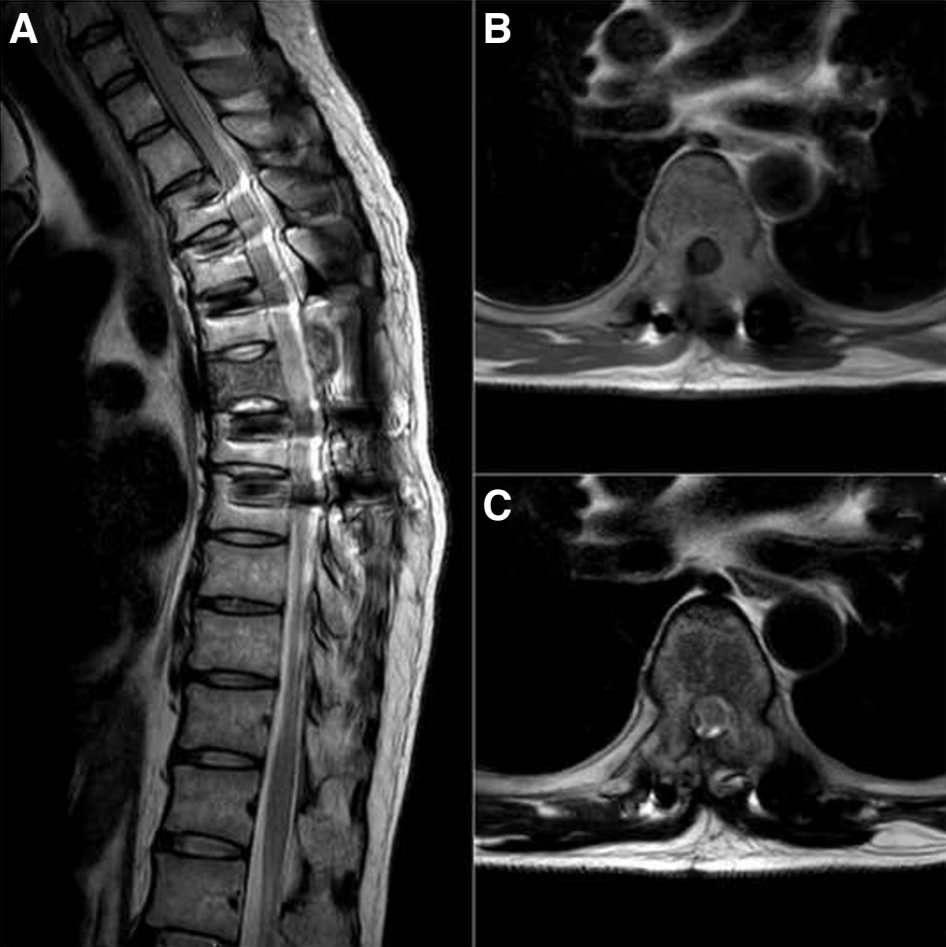
Repeat MRI at final follow-up showing resolution of the tumour. (A) T2 sagittal cut. (B) T1 axial cut. (C) T2 axial cut.

A second surgery at this stage was considered high risk and the decision was made to initiate concurrent site specific T4-T7 radiotherapy (30 Gy in 10 fractions) along with chemotherapy. Interestingly at the end of radiotherapy and four courses of chemotherapy (4 months post-op), there was significant clinical improvement of neurology and complete resolution of the soft tissue enhancement surrounding the spinal cord was evident in the MRI (Fig. 7). With appropriate physiotherapy and rehabilitation, patient gradually regained full power in both lower limbs by 6 months. He continues to be under oncology follow up and is ambulant with support.

## 3. Discussion

Metastatic NET involving the thoracic vertebra with epidural extension causing severe cord compression is an uncommon but aggressive disease [8]. It is a challenge to manage this form of tumour when it presents with metastasis at the time of primary diagnosis as in our patient. When metastatic spinal cord compression presents with neurological deficits, immediate decompression surgery is advised [9, 10]. However, this provided only short interval resolution of symptoms in our patient. Subsequently, the tumour recurred to a greater extent within a month highlighting the aggressive nature.

In such a scenario where early recurrence causes neurological deficits, there is a dilemma whether to perform revision surgery or to continue chemo-radiotherapy. We decided to choose the later considering the risk of revision surgery. We therefore initiated chemotherapy with Carboplatin and Etoposide which is the usual protocol for managing metastatic NETs [11–13]. In addition, targeted radiotherapy (30 Gy in 10 fractions) to T4-T7 vertebra was given to restrain local growth of the tumour and prevent further deterioration [14, 15]. In spite of an exuberant recurrence, the tumour was sensitive to chemo-radiotherapy and the patient improved symptomatically with dramatic improvement on follow-up MRI scans.

## 4. Conclusion

This case demonstrates a rare aggressive recurrence of a metastatic vertebral neuro-endocrine tumour causing severe cord compression in less than 1 month after surgery. While revision decompression is an option of treatment, this comes with a high surgical risk and a strong possibility for repeated recurrence. We would like to emphasize the importance of early chemo-radiother- apy following the index surgery as an effective treatment, even in circumstances where a recurrence has occurred, bypassing the risk of a revision surgery.
